# Decreased expression of BTG3 was linked to carcinogenesis, aggressiveness, and prognosis of ovarian carcinoma

**DOI:** 10.1007/s13277-013-0811-2

**Published:** 2013-05-09

**Authors:** Boya Deng, Yang Zhao, Wenfeng Gou, Shuo Chen, Xiaoyun Mao, Yasuo Takano, Huachuan Zheng

**Affiliations:** 1 Department of Gynecology, The First Affiliated Hospital of China Medical University, Shenyang, 110001 People’s Republic of China; 2 Department of Biochemistry and Molecular Biology, Institute of Pathology and Pathophysiology, College of Basic Medicine, China Medical University, Shenyang, 110001 People’s Republic of China; 3 Clinical Cancer Institute, Kanagawa Cancer Center, Yokohama, 241-0815 Japan

**Keywords:** Epithelial ovarian carcinoma, BTG3, Down-regulation, Prognosis, Pathological behavior

## Abstract

B-cell translocation gene 3 (BTG3) is a member of the BTG family which inhibits cell proliferation, metastasis, and angiogenesis, and also regulates cell-cycle progression and differentiation in a variety of cell types. However, there is no study to analyze BTG3 expression in epithelial ovarian carcinoma (EOC). Here, we investigated the expression of BTG3 in EOC carcinogenesis and subsequent progression. BTG3 mRNA expression was detected by real-time RT–PCR in ovarian benign and malignant tumors. The expression of BTG3 protein was examined by immunohistochemistry on tissue microarrays containing ovarian normal tissue, benign and borderline epithelial ovarian tumors, and EOCs. Relationships of BTG3 with both EOC clinicopathology and prognosis were analyzed statistically. The expression of BTG3 protein was also evaluated in ovarian normal tissue, benign tumors, and EOCs by western blot. The BTG3 mRNA expression level was higher in ovarian normal tissue and benign tumors than that in borderline, primary, and metastatic carcinoma (*p* < 0.05), and was negatively correlated with dedifferentiation and FIGO staging of EOC (*p* < 0.05). Using western blot, BTG3 protein was found lower in EOCs compared to the normal and benign tumors (*p* < 0.05), and poorly differentiated EOCs showed lower BTG3 expression than well-differentiated and moderately differentiated EOCs (*p* < 0.05). Immunohistochemically, BTG3 protein expression was statistically lower in EOCs than normal tissue and benign tumors (*p* < 0.05). EOC patients with low BTG3 protein expression showed a higher incidence of metastasis (*p* = 0.020), poor differentiation (*p* = 0.030), and shorter disease-free time and overall survival time (*p* < 0.05). By using Cox’s proportional hazard model, BTG3 protein expression and FIGO staging were independent prognostic factors for both disease-free time and overall survival time of EOCs (*p* < 0.05). It was suggested that down-regulated BTG3 expression might play roles in the pathogenesis and aggressiveness of EOC. BTG3 protein expression may be considered as a good marker to indicate the favorable prognosis of EOCs.

## Introduction

Ovarian cancer is the second leading cancer in women and the fifth leading cause of cancer-related deaths in women. Epithelial ovarian carcinoma (EOC) is disproportionately deadly because no sophisticated approach for the early diagnosis makes most ovarian cancers diagnosed at advanced stages [1]. Treatment may be shortly effective; patients with advanced International Federation of Gynecology and Obstetrics (FIGO) stages still have poor prognosis with 5-year survival rates below 33 % depending on response to chemotherapy [2]. EOCs are believed to arise from the ovarian epithelium and fallopian tubes, probably due to such risk factors as hereditary, reproductive, hormonal, inflammatory, hereditary, and geographic factors [3]. Carcinogenesis and progression of ovarian carcinoma are multistage processes, and increased understanding of the changes that occur in gene expression during carcinogenesis may result in the improvement of its diagnosis, treatment, and prevention. Therefore, it is an urgent need to identify more effective and new molecular targeted therapies for EOC.

The human BTG (B-cell translocation gene)/Transducer of ErbB2 gene family comprises of six proteins (BTG1, BTG2/TIS21/PC3, BTG3, BTG4/PC3B, Transducer of ErbB-2, and TOB2), which inhibit cell proliferation and regulate cell-cycle progression and differentiation in a variety of cell types. BTG is a nuclear protein that is imported into the nucleus through a nuclear localization signal (NLS)-mediated mechanism, and its nucleocytoplasmic translocation depends on cell growth states [4]. The conserved N-terminal BTG domain encompasses 104–106 amino acids and contains Box A and B. It has been reported that the conserved BTG domain mediates interactions with the highly similar Caf1a (CNOT7) and Caf1b (CNOT8) catalytic subunits of the Ccr4–Not deadenylase complex, which was closely associated with the anti-proliferative activity of BTG/TOB proteins. Furthermore, the activity of BTG/TOB proteins in the regulation of mRNA abundance and translation is dependent on Caf1a/Caf1b [5]. BTG3/ANA/APRO4 (MGC8928, Protein BTG3, Protein Tob5, TOB5, tob55, TOB55, TOFAD) is a member of the anti-proliferative BTG family and has been reported to be a tumor suppressor gene in some malignancies [6]. The human *BTG3* gene is localized in chromosome 21q21.1, and its two cDNA variants encode two variants with 132-nucleotide deletion by alternative splicing [7–9]. BTG3 is a downstream target of p53 and interacts with E2F1 to suppress the DNA binding of the E2F1–DP1 transcription factor complex through an N-terminal domain including the conserved Box A, suggesting its negative regulatory influence on cellular S-phase progression [10]. BTG3 is also able to interact with the Smad8 receptor-regulated Smad transcription factor as BTG1 and BTG2 [11]. BTG3 associates with Src via its C-terminal proline-rich domain to down-regulate Src tyrosine kinase activity and suppresses Ras/MAP kinase signaling. *BTG3* deficiency enhances bone morphogenetic protein-induced ectopic bone formation via transcriptional events [12]. BTG3 can associate with Caf1 and is a preferred partner of the CCR4 transcription factor-associated protein Caf1 by its amino-terminal half [13]. Loss of BTG3 in normal cells induced cellular senescence, which was correlated with enhanced ERK-AP1 signaling and elevated expression of the histone H3K27me3 demethylase JMJD3/KDM6B, leading to acute induction of p16(INK4a) [14].

In mice, *BTG3* mRNA was ubiquitously expressed in adult mice, the level being relatively high in the heart, lung, kidney, and testis, but low in the spleen and skeletal muscle. In human, BTG3 expression is down-regulated in lung, prostate, or renal cancer tissues and cells, and induced by genistein and 5-aza-2′-deoxycytidine, suggesting silenced BTG3 expression is attributable to its epigenetic methylation [8, 15–17]. Long-term observation of BTG3-deficient mice reveals that 8 % of them develop lung tumors (5/66) by 21 months after birth. Exogenous BTG3 protein suppresses the levels of matrix metalloproteinase-2 and plasminogen activator inhibitor-1 expression in lung cancer cells [15]. Taken together, it is suggested that BTG3 protein might have a negative regulatory effect on tumor progression by suppressing angiogenesis, invasion, and metastasis. In our previous work, aberrant BTG3 expression was found to link to gastric carcinogenesis and its venous invasion (unpublished). To explore the roles of *BTG3* expression in the ovarian carcinogenesis and subsequent progression, we examined the expression of BTG3 mRNA and protein in ovarian normal, benign, and borderline tumor, primary, and metastatic epithelial ovarian carcinoma in omentum, and compared them with clinicopathological parameters of EOCs.

## Materials and methods

### Tissue samples

Between January 2005 and December 2011, ovarian normal tissue, benign and borderline epithelial ovarian tumors (serous and mucinous types were included), primary epithelial ovarian carcinoma, and omentum with metastatic tumor were collected from surgical resection at the Department of Gynecology, The First Hospital Affiliated to China Medical University. The average age at surgery was 51.6 years (range 20–81 years). The parts of ovarian tissues were subjected to the routine preparation of pathological block. Some samples were frozen immediately in liquid nitrogen and stored at −80 °C until use. None of the patients underwent chemotherapy, radiotherapy, or adjuvant treatment before surgery. We followed up the patients by consulting their case documents and by telephone. Informed consents were obtained from all subjects, and the study was approved by the China Medical University Ethics Committee.

### Pathology

All tissues were fixed in 10 % neutral formalin, embedded in paraffin, and sections cut at 4 μm. These sections were stained by hematoxylin and eosin (HE) to confirm their histological diagnosis and other microscopic characteristics. The staging for each ovarian carcinoma was evaluated according to the International Federation of Gynecology and Obstetrics (FIGO) staging system for the extent of tumor spread. Histological architecture of ovarian carcinoma was expressed in terms of WHO classification.

### Real-time RT–PCR

Total RNA was extracted from ovarian tissues using QIAGEN RNeasy mini kit (QIAGEN, Germany). Two micrograms of total RNA was subjected to cDNA synthesis using the AMV transcriptase and random primers (Takara, Otsu, Japan). Oligonucleotide primers for PCR were sense, 5′-TGAAGTTA GATGGGCCAAAC-3′, and anti-sense, 5′-CCAACAGAGTTGATGCACAA-3′, for *BTG3* (NM_001130914.1, 1243–1425, 183 bp); and sense, 5′-CAATGACCCCTTCATTGACC-3′, and anti-sense, 5′-TGGAAG ATGGTGATGGGATT-3′, for GAPDH (135 bp, 201–335, NM_002046.3). PCR amplification of cDNA was performed in 20-μl mixtures according to the protocol of SYBR Premix Ex Taq^TM^ II kit (Takara).

### Western blot

Frozen tissues were washed twice with ice-cold PBS and homogenized on ice in 10 vol (w/v) of lysis buffer containing 20 mM Tris–HCl, 1 mM EDTA, 50 mM NaF, 50 mM NaCl, 1 mM Na_3_VO_4_, 1 % Triton X-100, and 1 mM PMSF. The homogenate was centrifuged at 15,000 rpm for 30 min at 4 °C. The supernatant was collected and stored at −70 °C. Protein content was determined using the BCA assay (Beyotime Institute of Biotechnology, Jiangsu, China). Protein was separated by 10 % SDS–PAGE and then transferred to PVDF blotting membranes, which were then blocked for 2 h in 5 % milk in Tris-buffered saline containing Tween-20 (TBST—10 mM Tris–HCl, 150 mM NaCl, and 0.1 % Tween-20). For immunoblotting, the membrane was incubated at 4 °C overnight with rabbit antibody against BTG3 (1:1,000) from Sigma (HPA018400; Sigma, USA) and mouse anti-β-actin antibody (1:1,000; Keygen Biotech, Nanjing, China). Then, it was rinsed with TBST three times and incubated with horseradish peroxidase conjugated anti-rabbit or anti-mouse IgG antibodies (1:2,000; Zhongshan Golden Bridge Biotechnology, Beijing, China) for 2 h. The Imaging System (DNR Bio-Imaging Systems, Israel) was used for image capture. The optical density (OD) of each band was measured using Image J software. The BTG3 and β-actin OD ratio was calculated as relative content and expressed graphically for the ovarian samples.

### Tissue microarray

Representative areas of solid tumors were identified in HE-stained sections of the selected tumor cases and a 2-mm-diameter tissue core per donor block was punched out and transferred to a recipient block with a maximum of 48 cores using a Tissue Microarrayer (AZUMAYA KIN-1, Japan). Four-micrometer-thick sections were consecutively incised from the recipient block and transferred to poly-l-lysine-coated glass slides. HE staining was performed on TMA for the confirmation of tumor tissue.

### Immunohistochemistry

Consecutive sections were deparaffinized with xylene, rehydrated with alcohol, and subjected to antigen retrieval by irradiating in target retrieval solution (TRS; DAKO, USA) for 15 min with a microwave oven. The sections were quenched with 3 % hydrogen peroxide in absolute methanol for 20 min to block endogenous peroxidase activity. Five percent bovine serum albumin was then applied for 5 min to prevent non-specific binding. The sections were incubated with the rabbit antibody against human BTG3 (1:100) from Sigma, anti-Ki-67 (DAKO, 1:50) for 15 min, then treated with the anti-rabbit conjugated to horseradish peroxidase (DAKO, USA; 1:00) antibodies for 15 min. All the incubations were performed in a microwave oven to allow intermittent irradiation as described previously [18]. After each treatment, the slides were washed with TBST three times for 1 min. Binding sites were visualized with 3, 3′-diaminobenzidine. After counterstained with Mayer’s hematoxylin, the sections were dehydrated, cleared, and mounted. Omission of the primary antibody was used as a negative control.

One hundred cells were randomly selected and counted from five representative fields of each section blindly by two independent observers (Deng BY and Zheng HC). The inconsistent data were confirmed by both persons until final agreements were reached. The expression positivity was graded and counted as follows: 0 = negative, 1 = 1–49 %, 2 = 50–74 %, and 3 ≥75 %. The staining intensity score was graded as follows: 1 = weak, 2 = intermediate, and 3 = strong. The scores for BTG3 positivity and staining intensity were multiplied to obtain a final score, which determines their expression as − = 0, + = 1–2, ++ = 3–4, and +++ = 6–9. The expression positivity of Ki-67 was graded and counted as follows: 0 = negative, 1 = 1–49 %, 2 = 50–74 %, and 3 ≥75 %.

### Measurement of CA125

Serum CA125 was determined by Quantitative Chemiluminescence Immunoassay Kit (Gentaur, France). Briefly, 50 μl of standard (0–1,000 U/ml), specimens, and controls were dispensed into appropriate wells. Then, we added 100 μl of Enzyme Conjugate Reagent into each well, gently mixed, and incubated the plate at room temperature for 60 min. The microtiter wells were rinsed and flicked with wash buffer. After that, residual water droplets were removed by striking the well sharply onto absorbent paper. Finally, 100 μl Chemiluminescence substrate solution into each well was dispensed, mixed gently, and subjected to absorbance determination.

### Statistical analysis

Statistical evaluation was performed using Spearman’s correlation test to analyze the rank data and Wilcoxon’s test to differentiate the means of different groups. Kaplan–Meier survival plots were generated and comparisons between survival curves were made with log-rank test. Cox’s proportional hazards model was employed for multivariate analysis. SPSS 10.0 software was applied to analyze all data, and *p* <0.05 was considered statistically significant.

## Results

### The correlation of BTG3 mRNA expression with tumorigenesis and clinicopathological features of ovarian carcinoma

**Fig. 1:**
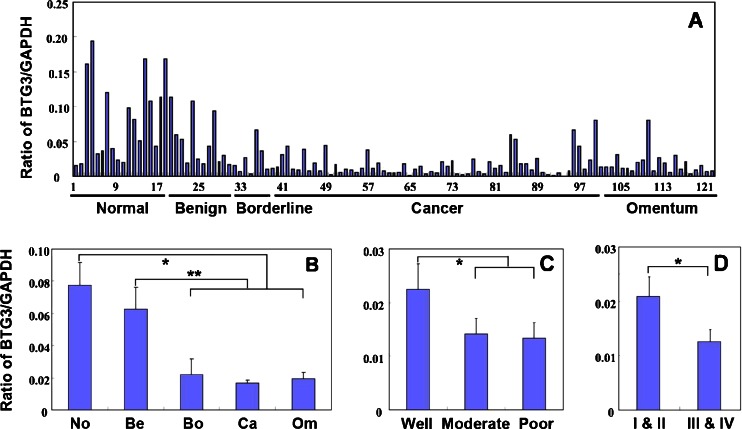
The correlation of *BTG3* mRNA expression with tumorigenesis and aggressive features of ovarian carcinoma. Real-time RT–PCR was employed to amplify *BTG3* mRNA in 121 ovarian samples (**a**). *BTG3* mRNA level was higher in ovarian normal tissue (*No*, *n* = 17) and benign tumor (*Be*, *n* = 12) than that in borderline tumor (*Bor*, *n* = 6), primary carcinoma (*Ca*, *n* = 65), or metastatic carcinoma in omentus (*Om*, *n* = 21) (*p* < 0.05, **b**). No statistical differences were found between ovarian normal tissue (*No*) and benign tumor (*Be*), or among borderline tumor (*Bor*), primary carcinoma (*Ca*), or metastatic carcinoma in omentus (*Om*). Well-differentiated carcinoma showed higher *BTG3* mRNA expression in comparison with moderately and poorly differentiated ones (*p* < 0.05, **c**). *BTG3* mRNA displayed lower expression in advanced FIGO staging EOC (*p* < 0.05, **d**)

We measured the expression of BTG3 mRNA in ovarian normal tissue (*n* = 17), benign tumor (*n* = 12), borderline tumor (*n* = 6), and primary (*n* = 65) and metastatic carcinoma (*n* = 21) by real-time RT–PCR. As shown in Fig. 1, BTG3 mRNA expression level was higher in ovarian normal tissue and benign tumor, and lower in borderline tumor and primary and metastatic carcinoma (*p* < 0.05, Fig. 1a, b). Well-differentiated carcinoma showed higher BTG3 mRNA expression in comparison with moderately and poorly differentiated ones (*p* < 0.05, Fig. 1c). BTG3 mRNA displayed lower expression in advanced EOCs (FIGO III and IV) than FIGO I and II staging EOC (*p* < 0.05, Fig. 1d).

### BTG3 protein expression during ovarian carcinogenesis and its correlation with clinicopathological parameters of ovarian carcinoma

**Fig. 2:**
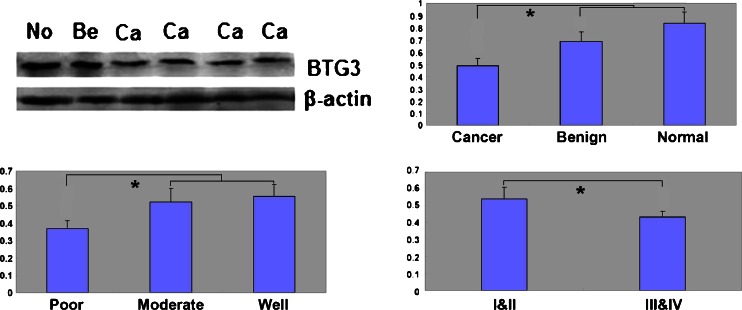
The correlation of BTG3 protein expression with tumorigenesis and aggressive features of ovarian carcinoma. Western blot was employed to valuate BTG3 protein expression in 78 ovarian samples (**a**). BTG3 was higher in ovarian normal tissue (*n* = 9) and benign tumor (*n* = 10) than that in EOCs (*n* = 59) (*p* < 0.05, **b**). Well-differentiated and moderately differentiated EOC showed higher BTG3 expression in comparison with poorly differentiated ones (*p* = 0.045, **c**). No statistical difference of BTG3 expression was found between FIGO I/II and FIGO III/IV staging (*p* > 0.05, **d**)

**Fig. 3:**
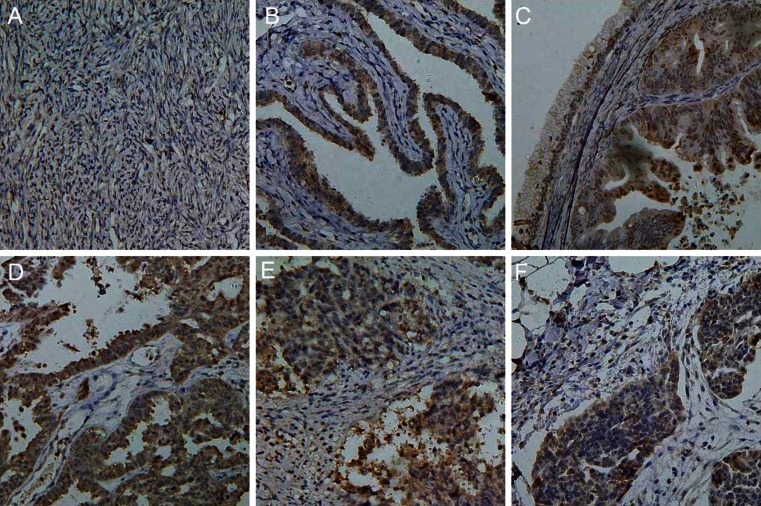
Immunohistochemical staining of BTG3 protein in ovarian samples. BTG3 protein was positively detected in the cytoplasm of ovarian fiber cells (**a**), fallopian tube (**b**), borderline serous tumor (**c**), primary serous adenocarcinoma (**d**, **e**), and metastatic serous adenocarcinoma in omentus (**f**)

By western blot assays, we measured BTG3 protein expression in normal ovary tissues (*n* = 9), benign ovarian tumors (*n* = 10), and EOCs (*n* = 59). BTG3 was lower in EOCs than in normal ovary tissues (*p* = 0.002) and benign ovarian tumors (*p* = 0.046) (Fig. 2). BTG3 was lower in poorly differentiated EOCs than well-differentiated and moderately differentiated EOCs (*p* = 0.045) (Fig. 2b). As shown in Fig. 3, BTG3 protein distributed in the cytoplasm of normal ovary tissue, ovarian benign tumor, and EOCs. BTG3 expression was detectable in normal ovary tissue (78.6 %, 22/28), benign tumor (80.0 %, 12/15), borderline tumor (41.7 %, 10/24), carcinoma (51.0 %, 127/249), and metastatic carcinoma in omentum (50.0 %, 24/48), respectively. According to its frequency and density, BTG3 protein expression was statistically lower in ovarian carcinoma than normal ovary and benign tumor (*p* < 0.05, Table 1). Its expression level was lower in ovarian borderline tumors than normal tissue (*p* < 0.05, Table 1). As summarized in Table 2, BTG3 expression was lower in EOCs of advanced FIGO staging (*p* = 0.020), moderate or poor differentiation EOC (*p* = 0.030), and higher Ki-67 expression (*p* = 0.042). BTG3 expression in EOCs was not associated with the age of the patients, serum CA125, or pathological classification (*p* > 0.05).

**Table 1:** BTG3 expression in ovarian carcinogenesis

Groups	*n*	BTG3 expression				
		−	+	++	+++	*p* value
Normal ovary	28	6	4	5	13	
Ovarian benign tumor	15	3	1	4	7	
Ovarian borderline tumor	24	14	4	2	4	0.037*
Ovarian carcinoma	249	122	44	50	33	0.008*
						0.022**
Metastatic carcinoma in omentus	48	24	12	8	4	0.002*
						0.001**

*p < 0.05, compared with ovarian normal tissue; **p < 0.05, compared with benign tumors

**Table 2:** The relationship between BTG3 expression and clinicopathological features of ovarian carcinomas

Clinicopathological features	*n*	BTG3 expression					
		−	+	++	+++	PR (%)	*p* value
Age (years)							0.466
<56	123	61	25	24	13	47.2	
≥56	126	61	19	26	20	54.8	
Pathological classification							0.715
Serous adenocarcinoma	185	95	29	36	25	48.6	
Mucinous adenocarcinoma	26	11	5	7	3	57.7	
Miscellaneous subtypes	38	16	10	7	5	57.8	
FIGO staging							0.020
I–II	93	34	18	28	13	63.4	
III–IV	156	88	26	22	20	43.6	
Differentiation							0.030
Well differentiated	60	19	17	16	8	68.3	
Moderately differentiated	103	53	14	19	17	48.5	
Poorly differentiated	86	50	13	15	8	41.9	
Serum CA125 concentration (U/ml)							0.085
<500	45	20	3	12	10	44.4	
≥500	54	26	16	9	3	51.9	
Ki-67 expression							0.042
−	54	15	18	14	7	72.2	
+	38	25	7	3	3	34.2	
++	35	19	3	7	6	45.7	
+++	39	28	1	5	5	28.2	

### Survival analysis

**Fig. 4:**
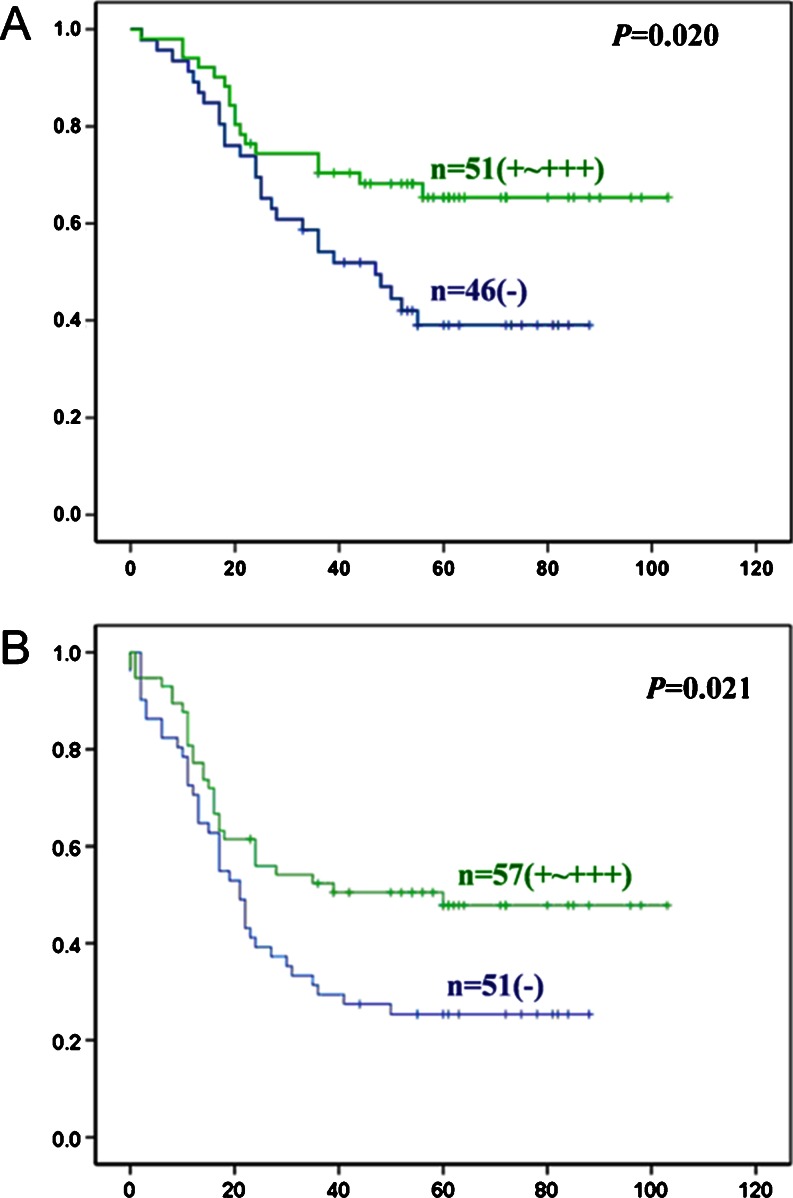
Correlation between BTG3 protein expression status and prognosis of ovarian carcinoma patients. Kaplan–Meier curves for overall (**a**) or disease-free (**b**) survival rate of patients with ovarian carcinomas according to BTG3 protein expression. It was indicated that patients with lower BTG3 expression have more likely shorter overall survival and disease-free time (overall survival time, *p* = 0.020; disease-free time, *p* = 0.021)

Follow-up information was available on 108 ovarian carcinoma patients for periods ranging from 1 to 107 months (median=62.3 months). Survival curves for EOC were stratified according to BTG3 expression (Fig. 4). By using the Kaplan–Meier method, we indicated that both survival time and disease-free time for patients were linked to BTG3 expression status (overall survival time, *p* = 0.020, Fig. 4a; disease-free time, *p* = 0.021, Fig. 4b). Multivariate analysis using Cox’s proportional hazard model indicated that FIGO staging and BTG3 protein expression (*p* < 0.05) but not the patient age, pathological classification, differentiation residual lesion size, or serum CA125 level were independent prognostic factors for overall survival of EOC (*p* > 0.05, Table 3). Further, the patient’s age, FIGO staging, and BTG3 protein expression (*p* < 0.05) but not pathological classification, differentiation residual lesion size, or serum CA125 level were independent prognostic factors for disease-free ovarian carcinomas (*p* > 0.05, Table 4).

**Table 3:** Multivariate analysis of clinicopathological variables for the overall survival of the patients with ovarian carcinomas

Clinicopathological parameters	Relative risk (95% CI)	*p* value
Age (≥56 years)	1.891 (0.965–3.704)	0.063
Pathological classification (serous adenocarcinoma)	0.711 (0.275–1.837)	0.481
FIGO staging (I–II)	3.971 (1.675–9.416)	0.002
Differentiation (poor)	1.810 (0.905–3.620)	0.093
Residual lesion size (≥1 cm)	1.299 (0.628–2.689)	0.481
Serum CA125 concentration (≥500 U/ml)	0.632 (0.328–1.220)	0.172
BTG3 expression (+ to +++)	0.352 (0.181–0.688)	0.002

**Table 4:** Multivariate analysis of clinicopathological variables for the relapse-free survival of the patients with ovarian carcinomas

Clinicopathological parameters	Relative risk (95% CI)	*p* value
Age (≥56 years)	1.786 (1.043–3.059)	0.035
Pathological classification (serous adenocarcinoma)	0.636 (0.302–1.343)	0.235
FIGO staging (I–II)	2.105 (1.094–4.052)	0.026
Differentiation (poor)	1.265 (0.722–2.216)	0.412
Residual lesion size (≥1 cm)	1.264 (0.698–2.291)	0.439
Serum CA125 concentration (≥500 U/ml)	0.962 (0.569–1.626)	0.885
BTG3 expression (+ to +++)	0.455 (0.272–0.726)	0.003

## Discussion

Increasing evidence suggests that BTG3 is thought to be a negative regulator of cellular S-phase progression, and it has been shown that its anti-proliferative action is through inhibition of transcription factor E2F1 or interaction with Smad transcription factor as a tumor suppressor [10, 11]. Here, we for the first time examined in situ BTG3 expression in ovarian normal tissue, benign and borderline tumor, and carcinoma samples. It was found that BTG3 protein was mainly localized in the cytoplasm of ovarian fiber cells, fallopian tube, benign and borderline tumor, and carcinoma cells. It was suggested that the BTG3 expression pattern has cellular specificity, which determines its biological functions. However, the mechanisms of its cell-specific characteristics should be further investigated.

Using western blot and immunochemistry staining, BTG3 expression was reduced in ovarian carcinoma, compared with ovarian normal tissue and benign tumor, indicating that down-regulated BTG3 expression contributes to ovarian epithelial carcinogenesis. In the present study, *BTG3* mRNA level was reduced from ovarian borderline tumor and carcinoma, in line with the immunohistochemical data. Yoneda et al. [15] also reported that *BTG3* mRNA expression was down-regulated in lung carcinoma tissue or cell lines, consistent with the normal counterparts. Yu et al. [8] demonstrated that *BTG3* expression was markedly reduced and its promoter region of *BTG3* was hypermethylated without detectable mutations in the promoter and coding region in a wide variety of human breast cancer cell lines, suggesting that hypermethylation might be an important mechanism for inactivation of BTG3. Methylation-mediated down-regulation of *BTG3* was also documented in renal and prostate cancer cells [16, 17]. Sasajima et al. [19] suggested that anti-proliferative proteins of the BTG/Tob family might be degraded by the ubiquitin–proteasome system. The ubiquitin-mediated degradation of BTG protein could support the BTG3 hypoexpression in ovarian borderline tumor and EOCs. However, the mechanism of BTG3 down-regulation in EOC will be investigated in the future study.

In the present study, BTG3 protein expression was positively linked to clinicopathological features of ovarian carcinoma, including differentiation and FIGO staging in agreement with the data of our real-time RT–PCR, indicating that BTG3 might be involved in the development and differentiation of EOC and be considered as a good biomarker to indicate the aggressive behaviors of EOC. We also compared the expression of BTG3 and Ki-67 in EOC samples. Ki-67 is a nuclear protein found in G1 phase of cell cycle associated with cell proliferation. As an excellent marker to determine the cell proliferation index, Ki-67 was also associated with EOC progression and prognosis [20]. Since increased Ki-67 expression revealed an increase in mitotic cell activity and proliferation, the negative correlation we found between BTG3 and Ki-67 expression provided evidence about the suppressive effects of BTG3 on the cell proliferation. Regarding the prognostic significance of other *BTG*s, Möllerström et al. [21] demonstrated high-level BTG2 protein expression correlates with prolonged survival in patients with breast carcinoma. Kamalakaran et al. [22] identified differential methylation of CpG islands proximal to BTG1 as having prognostic value independent of subtypes and other clinical factors of luminal breast cancers. We revealed the inverse links between BTG3 expression levels and the overall and disease-free survival of patients with EOC in the present study. Multivariate analysis demonstrated that BTG3 protein expression was an independent factor to indicate the favorable prognosis of ovarian carcinoma. Also in our study, FIGO staging was the independent factor of both the overall and disease-free survival of the EOC, as other researchers revealed [23, 24]. Since Bast et al. [25] had developed an assay for serum CA125 and found it elevated in 82 % of EOC patients, serum CA125 kinetics during chemotherapy had value in predicting survival of EOCs. Some research also revealed that pre-operative serum CA125 was an independent factor to EOC prognosis [26]. But in our study, there is no statistical relationship between pre-operative CA125 and prognosis of EOCs. This may be due to many characteristics which could profoundly influence serum CA125, including tumor characteristics such as histology, grade, stage, and presence of ascites together with certain conditions such as peritoneal and mucosal inflammation [27]. Our findings suggested that BTG3 protein expression could be employed to indicate the prognosis of ovarian carcinoma patients as an independent factor.

In conclusion, our study indicated that BTG3 was down-regulated in epithelial ovarian carcinoma, which might have impact on the clinicopathogenesis of EOC, and should be considered as a good biomarker for ovarian carcinogenesis and subsequent progression. Nevertheless, the biological functions of BTG3 in EOC need further investigation.

## References

[1] Jemal A, Siegel R, Ward E (2007). Cancer statistics, 2007. CA Cancer J Clin.

[2] Heintz APM, Odicino F, Maisonneuve P (2006). Carcinoma of the ovary. Int J Gynecol Obstet.

[3] Hunn J, Rodriguez GC (2012). Ovarian cancer: etiology, risk factors, and epidemiology. Clin Obstet Gynecol.

[4] Kawamura-Tsuzuku J, Suzuki T, Yoshida Y (2004). Nuclear localization of Tob is important for regulation of its antiproliferative activity. Oncogene.

[5] Doidge R, Mittal S, Aslam A (2012). The anti-proliferative activity of BTG/TOB proteins is mediated via the Caf1a (CNOT7) and Caf1b (CNOT8) deadenylase subunits of the Ccr4–Not complex. PLoS One.

[6] Winkler GS (2010). The mammalian anti-proliferative BTG/Tob protein family. J Cell Physiol.

[7] Guéhenneux F, Duret L, Callanan MB (1997). Cloning of the mouse BTG3 gene and definition of a new gene family (the BTG family) involved in the negative control of the cell cycle. Leukemia.

[8] Yu J, Zhang Y, Qi Z (2008). Methylation-mediated downregulation of the B-cell translocation gene 3 (BTG3) in breast cancer cells. Gene Expr.

[9] Yamamoto N, Uzawa K, Yakushiji T (2001). Analysis of the ANA gene as a candidate for the chromosome 21q oral cancer susceptibility locus. Br J Cancer.

[10] Ou YH, Chung PH, Hsu FF (2007). The candidate tumor suppressor BTG3 is a transcriptional target of p53 that inhibits E2F1. EMBO J.

[11] Miyai K, Yoneda M, Hasegawa U (2009). ANA deficiency enhances bone morphogenetic protein-induced ectopic bone formation via transcriptional events. J Biol Chem.

[12] Rahmani Z (2006). APRO4 negatively regulates Src tyrosine kinase activity in PC12 cells. J Cell Sci.

[13] Yoshida Y, Hosoda E, Nakamura T (2001). Association of ANA, a member of the antiproliferative Tob family proteins, with a Caf1 component of the CCR4 transcriptional regulatory complex. Jpn J Cancer Res.

[14] Lin TY, Cheng YC, Yang HC (2012). Loss of the candidate tumor suppressor BTG3 triggers acute cellular senescence via the ERK–JMJD3–p16(INK4a) signaling axis. Oncogene.

[15] Yoneda M, Suzuki T, Nakamura T (2009). Deficiency of antiproliferative family protein Ana correlates with development of lung adenocarcinoma. Cancer Sci.

[16] Majid S, Dar AA, Shahryari V (2010). Genistein reverses hypermethylation and induces active histone modifications in tumor suppressor gene B-cell translocation gene 3 in prostate cancer. Cancer.

[17] Majid S, Dar AA, Ahmad AE (2009). BTG3 tumor suppressor gene promoter demethylation, histone modification and cell cycle arrest by genistein in renal cancer. Carcinogenesis.

[18] Kumada T, Tsuneyama K, Hatta H (2004). Improved 1-h rapid immunostaining method using intermittent microwave irradiation: practicability based on 5 years application in Toyama Medical and Pharmaceutical University Hospital. Mod Pathol.

[19] Sasajima H, Nakagawa K, Yokosawa H (2002). Antiproliferative proteins of the BTG/Tob family are degraded by the ubiquitin–proteasome system. Eur J Biochem.

[20] Liu P, Sun Y-L, Du J (2012). CD105/Ki67 coexpression correlates with tumor progression and poor prognosis in epithelial ovarian cancer. Int J Gynecol Cancer.

[21] Möllerström E, Kovács A, Lövgren K (2010). Up-regulation of cell cycle arrest protein BTG2 correlates with increased overall survival in breast cancer, as detected by immunohistochemistry using tissue microarray. BMC Cancer.

[22] Kamalakaran S, Varadan V, Giercksky Russnes HE (2011). DNA methylation patterns in luminal breast cancers differ from non-luminal subtypes and can identify relapse risk independent of other clinical variables. Mol Oncol.

[23] Kosary CL (1994). FIGO stage, histology, histologic grade, age and race as prognostic factors in determining survival for cancers of the female gynecological system: an analysis of 1973–87 SEER cases of cancers of the endometrium, cervix, ovary, vulva, and vagina. Semin Surg Oncol.

[24] Chen TH, Jansen L, Gondos A (2013). Survival of ovarian cancer patients in Germany in the early 21st century: a period analysis by age, histology, laterality, and stage. Eur J Cancer Prev.

[25] Bast RC, Klug TL, St John E (1983). A radioimmunoassay using a monoclonal antibody to monitor the course of epithelial ovarian cancer. N Engl J Med.

[26] Cooper BC, Sood AK, Davis CS (2002). Preoperative CA 125 levels: an independent prognostic factor for epithelial ovarian cancer. Obstet Gynecol.

[27] Cramer DW, Vitonis AF, Welch WR (2010). Correlates of the preoperative level of CA125 at presentation of ovarian cancer. Gynecol Oncol.

